# Mitochondrial Changes in Platelets Are Not Related to Those in Skeletal Muscle during Human Septic Shock

**DOI:** 10.1371/journal.pone.0096205

**Published:** 2014-05-01

**Authors:** Alessandro Protti, Francesco Fortunato, Maria L. Caspani, Mauro Pluderi, Valeria Lucchini, Nadia Grimoldi, Luigi P. Solimeno, Gigliola Fagiolari, Patrizia Ciscato, Samis M. A. Zella, Maurizio Moggio, Giacomo P. Comi, Luciano Gattinoni

**Affiliations:** 1 U.O. Terapia Intensiva “Emma Vecla”, Fondazione IRCCS Ca’ Granda – Ospedale Maggiore Policlinico, Università degli Studi di Milano, Milan, Italy; 2 U.O. Neurologia – Centro Dino Ferrari – Fondazione IRCCS Ca’ Granda – Ospedale Maggiore Policlinico, Università degli Studi di Milano, Milan, Italy; 3 U.O. Neurochirurgia, Fondazione IRCCS Ca’ Granda – Ospedale Maggiore Policlinico, Università degli Studi di Milano, Milan, Italy; 4 U.O. Malattie Neuromuscolari – Centro Dino Ferrari – Fondazione IRCCS Ca’ Granda – Ospedale Maggiore Policlinico, Università degli Studi di Milano, Milan, Italy; 5 U.O. Traumatologia, Fondazione IRCCS Ca’ Granda – Ospedale Maggiore Policlinico, Università degli Studi di Milano, Milan, Italy; D’or Institute of Research and Education, Brazil

## Abstract

Platelets can serve as general markers of mitochondrial (dys)function during several human diseases. Whether this holds true even during sepsis is unknown. Using spectrophotometry, we measured mitochondrial respiratory chain biochemistry in platelets and triceps brachii muscle of thirty patients with septic shock (within 24 hours from admission to Intensive Care) and ten surgical controls (during surgery). Results were expressed relative to citrate synthase (CS) activity, a marker of mitochondrial density. Patients with septic shock had lower nicotinamide adenine dinucleotide dehydrogenase (NADH)/CS (p = 0.015), complex I/CS (p = 0.018), complex I and III/CS (p<0.001) and complex IV/CS (p = 0.012) activities in platelets but higher complex I/CS activity (p = 0.021) in triceps brachii muscle than controls. Overall, NADH/CS (r^2^ = 0.00; p = 0.683) complex I/CS (r^2^ = 0.05; p = 0.173), complex I and III/CS (r^2^ = 0.01; p = 0.485), succinate dehydrogenase (SDH)/CS (r^2^ = 0.00; p = 0.884), complex II and III/CS (r^2^ = 0.00; p = 0.927) and complex IV/CS (r^2^ = 0.00; p = 0.906) activities in platelets were not associated with those in triceps brachii muscle. In conclusion, several respiratory chain enzymes were variably inhibited in platelets, but not in triceps brachii muscle, of patients with septic shock. Sepsis-induced mitochondrial changes in platelets do not reflect those in other organs.

## Introduction

Sepsis can be complicated by failure of organs distant from the site of infection, possibly because of a defect in oxygen-dependent mitochondrial energy production [1]. Underlying mechanisms may include hypoxia [2], [3] and inhibition of mitochondrial respiratory chain (where oxygen is normally used to generate adenosine triphosphate) [4].

Skeletal muscle is the tissue most commonly sampled for measuring mitochondrial biochemistry, even in critically ill patients [5]–[9]. It is readily accessible and can be examined according to well-standardized protocols [10]. During sepsis, skeletal muscle mitochondrial biochemistry possibly changes in line with that of other vital organs (liver, for instance [11]). However, muscle biopsy is an invasive procedure.

Studying platelets isolated from blood may be a valid alternative, with virtually no side effects. Platelets can serve as general markers of mitochondrial changes during neurologic diseases, senescence, diabetes and drug intoxication [12]–[17]. Whether this holds true during human sepsis remains unknown.

Aim of the present study was to compare platelet mitochondrial respiratory chain biochemistry with that of skeletal muscle in patients with septic shock.

## Materials and Methods

The study was approved by local Ethics Committee (Comitato Etico Ospedale Maggiore Policlinico, Mangiagalli e Regina Elena, Milano, Italy; protocol number 2724 – 18.10.2007) and registered at ClinicalTrials.gov (NCT00541827).

We enrolled thirty adults admitted to Intensive Care Unit (ICU) with septic shock [18] who had not received platelets within the last fifteen days and had not severe thrombocytopenia (<20000 platelets/mm^3^), coagulopathy (international normalized ratio >2.0) or known mitochondrial disease.

Within 24 h from admission, venous blood (15 ml) was drawn, anticoagulated with EDTA and sedimented for 45 min at 4°C. The top three-quarters of platelet-rich-plasma were centrifuged at 5000 g for 10 min. Pellet was washed with distilled water, centrifuged at 14500 g for 10 min, washed again with PBS and finally stored at −80°C (for mitochondrial biochemistry). Electron microscopy performed on three samples confirmed that pellet obtained in this way was formed almost entirely (≥80%) of platelets. Thereafter, three tissue fragments (≈0.4 cm^3^ each) were surgically taken from the right or left triceps brachii muscle, under general anaesthesia, and kept in ice. Following removal of blood, fat and connective tissue, samples were stored in liquid nitrogen (for mitochondrial biochemistry – see also Table S1), cooled isopentane (for histology and histochemistry) or 2.5% glutaraldehyde in PBS (pH 7.4) (for electron microscopy). The entire protocol (including contralateral biopsy) was repeated on day seven if patients were still in ICU.

Ten patients undergoing elective arm surgery (shoulder replacement or implant removal) under general anaesthesia acted as controls.

Written informed consent was obtained from septic patients when they returned conscious (deferred consent) and from surgical patients before surgery.

### Mitochondrial Biochemistry (Platelets and Skeletal Muscle)

Platelet pellet was diluted in buffer (KCl 120 mmol/l, HEPES 20 mmol/l, MgCl_2_ 5 mmol/l and EGTA 1 mmol/l; pH 7.2) (300–400 µl), sonicated (two cycles at 60 W for 10 sec) and centrifuged (750 g for 10 min) at 4°C. Skeletal muscle was diluted in the same buffer (1∶10), homogenized (three cycles at 350 g for 1 min) and centrifuged as above. Activities of nicotinamide adenine dinucleotide dehydrogenase (NADH) (subunit of complex I), NADH-ubiquinone 1 reductase (complex I), NADH-cytochrome *c* reductase (complex I+III), succinate dehydrogenase (SDH) (subunit of complex II), SDH-cytochrome *c* reductase (complex II+III) and cytochrome *c* oxidase (complex IV), main components of the mitochondrial respiratory chain, were measured on supernatants with spectrophotometry at 30°C [19]. Results are expressed relative to citrate synthase activity, a marker of mitochondrial density [6]–[9], [11], [15], [16].

### Histology and Histochemistry (Skeletal Muscle Only)

Biopsies were evaluated according to standard protocols [10], [20]. Inflammation was evaluated based on general examination, acid phosphatase staining, surface membrane binding of antibodies against human-leukocyte-antigen (HLA) class I and deposition of membrane attack complex (MAC). Glycogen was stained with Periodic-Acid-Schiff (PAS) method. Complex IV activity was judged abnormal if the proportion of negative fibres at double (complex IV and SDH)-labelling was ≥5‰. Defects were graded as minor (5–19‰), mild (20–39‰) or moderate (≥40‰).

### Electron Microscopy (Skeletal Muscle Only)

Three random biopsies of patients with septic shock (including one non-survivor) were post-fixed in 1% osmium tetroxide and embedded in Epon. Ultra-thin sections were counterstained with uranyl acetate and lead citrate. Mitochondrial ultrastructure was assessed with ZEISS EM-109.

### Statistical Analysis

Data collected from same patients on day one and seven were analyzed independently. Results are presented as means (±SD) or medians (IQR). Analysis was performed using Student’s *t* or Wilcoxon rank sum tests, one-way analysis of variance (ANOVA) or ANOVA on ranks (Holm-Sidak or Dunn’s method for post-hoc multiple comparisons). Strength of association between variables (r^2^) was assessed with Pearson product moment test. Proportions were compared using Fisher’s exact test. p<0.05 indicated statistical significance (SigmaPlot 11.0, Jandel Scientific Software; San Jose, CA, USA).

## Results

Thirty patients with septic shock were studied on day one (Table 1). They were older (68±12 *vs.* 54±12 years of age; p = 0.020) but similar for sex (17 males and 13 females *vs.* 6 males and 4 females; p = 1.000) to controls. Twenty patients were studied again on day seven.

**Table 1:** Main characteristics of patients with septic shock at ICU admission.

n	30
Surgical/Medical	21/9
Source of infection	
Abdomen	19
Lung	10
Central venous line	1
SAPS II	45 (40–53)
SOFA score	
Respiration	3 (3–3)
Coagulation	1 (0–1)
Liver	1 (0–2)
Cardiovascular	4 (4–4)
Central nervous system	0 (0–0)
Renal	1 (0–2)
Total	9 (9–11)
Central (or mixed) venous oxygen saturation (%)	76 (66–79)
Blood lactate (mmol/l)	3 (2–6)
Norepinephrine equivalent dose (µg/min)	15 (12–23)
On mechanical ventilation (n)	29
On renal replacement therapy (n)	3
Days from Hospital to ICU admission	1 (0–5)
Hours from ICU admission to study enrolment	20 (13–22)
Length of stay in ICU (days)	12 (5–18)
Discharged alive from Hospital (n)	25

ICU: intensive care unit. SAPS II: simplified acute physiology score II (referred to the first 24 h from admission). SOFA: sepsis-related organ failure assessment (referred to the first 24 h from admission). Norepinephrine equivalent dose was calculated as norepinephrine (µg/min)+[dopamine (µg/kg/min)÷2]+epinephrine (µg/min)+[phenylephrine (µg/min)÷10] [41]. Central venous oxymetry was monitored in twenty-five patients; mixed venous oxymetry was monitored in five patients. Please note that most of the patients were firstly resuscitated in the Emergency Department and then transferred to the ICU.

### Platelet Mitochondrial Biochemistry

**Figure 1:**
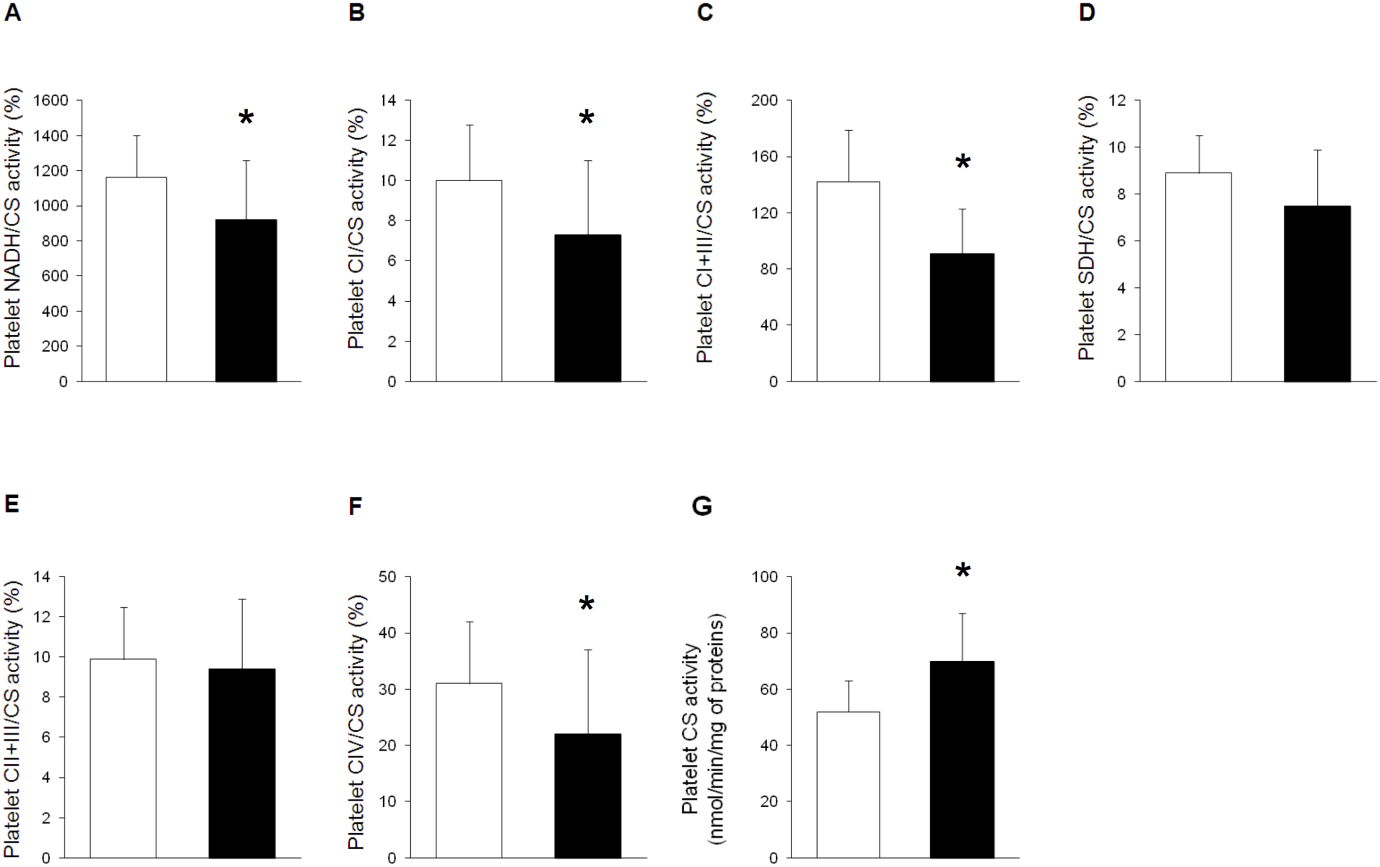
Platelet mitochondrial biochemistry during septic shock. Mitochondrial biochemistry was measured on platelets of ten surgical controls (white bars) and thirty patients with septic shock (<24 h from ICU admission) (black bars). Activities of nicotinamide adenine dinucleotide dehydrogenase (NADH) (p = 0.015^#^) (**A**), complex I (CI) (p = 0.018^#^) (**B**), complex I and III (CI+III) (p<0.001) (**C**), succinate dehydrogenase (SDH) (p = 0.086) (**D**), complex II and III (CII+III) (p = 0.672) (**E**) and complex IV (CIV) (p = 0.012^#^) (**F**) are expressed as percentages of citrate synthase (CS) activity (p = 0.002) (**G**). Data are reported as means and standard deviations. *p<0.05 *vs.* controls [Student’s *t* or (^#^) Wilcoxon rank sum tests]. Platelet count (182±83 *vs.* 176±63 *10^3^/mm^3^) did not differ between groups (p = 0.901).

On day one, platelets of patients with septic shock had lower NADH, complex I, complex I and III and complex IV (relative to citrate synthase) activities and higher citrate synthase activity than controls (Fig. 1). Inhibition of respiratory chain enzymes was apparently more severe in patients with higher sepsis-related organ failure assessment (SOFA) score, in those with thrombocytopenia and in non-survivors (Tables S2–S4). However, when exact values of SOFA score or platelet count were considered, their association with platelet mitochondrial biochemistry was usually non-significant (Fig. S1 and S2). On day seven, platelets of septic patients still had lower NADH and complex I (relative to citrate synthase) activities and higher citrate synthase activity than controls (Table S5).

### Skeletal Muscle Mitochondrial Biochemistry

**Figure 2:**
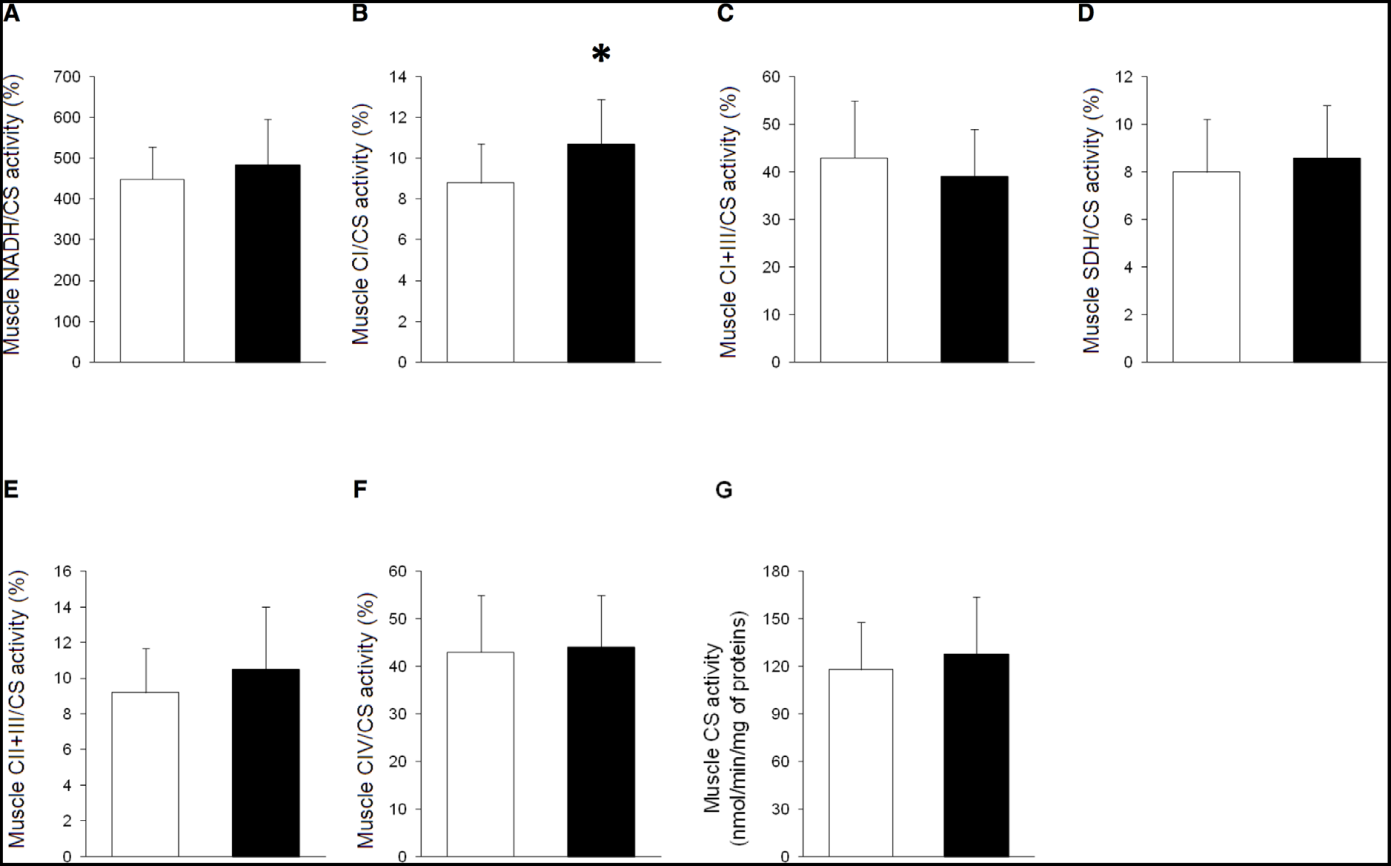
Skeletal muscle mitochondrial biochemistry during septic shock. Mitochondrial biochemistry was measured on triceps brachii muscle of ten surgical controls (white bars) and thirty patients with septic shock (<24 h from ICU admission) (black bars). Results of two patients are not available due to technical troubles. Activities of nicotinamide adenine dinucleotide dehydrogenase (NADH) (p = 0.369) (**A**), complex I (CI) (p = 0.021) (**B**), complex I and III (CI+III) (p = 0.286) (**C**), succinate dehydrogenase (SDH) (p = 0.432) (**D**), complex II and III (CII+III) (p = 0.273) (**E**) and complex IV (CIV) (p = 0.760) (**F**) are expressed as percentages of citrate synthase (CS) activity (p = 0.458) (**G**). Data are reported as means and standard deviations. *p<0.05 *vs.* controls (Student’s *t* test).

Skeletal muscle mitochondrial biochemistry of patients with septic shock (even the sickest ones) did not differ from that of controls, either on day one or seven (Fig. 2 and Tables S5–S9). The only exception was complex I (relative to citrate synthase) activity, that was initially higher (and not lower) in septic patients than in controls.

### Skeletal Muscle Histology and Histochemistry

On day one, few necrotic or regenerating fibres were noted in two (7%) and one (3%) patients. Acid phosphatase staining was clearly positive around blood vessels in five (17%) patients and in interstitium in four (13%) patients. Antibodies against HLA class I diffusely bound to membrane surface of two (7%) patients. Microvascular deposits of MAC were detected in one (3%) patient. PAS stain showed a generalized loss of glycogen in fourteen (47%) patients. Few fibres devoid of complex IV (but with normal succinate dehydrogenase) activity were noted in eight (27%) patients: defects were always minor except for one, mild, case. Other aspects of fibre morphology, vacuole density, vessels and connective tissue were unaltered. On day seven, notes of necrosis were found in four (out of eighteen) (22%) patients. Defects of complex IV activity were minor in two (11%) and mild in three (17%) cases. Other aspects were as above. Findings in controls were normal.

### Skeletal Muscle Electron Microscopy

Electron microscopy did not reveal any significant change. Mitochondria were typically located near the Z line and had classical morphology (Fig. S3).

### Relationship between Platelet and Skeletal Muscle Mitochondrial Biochemistry

**Figure 3:**
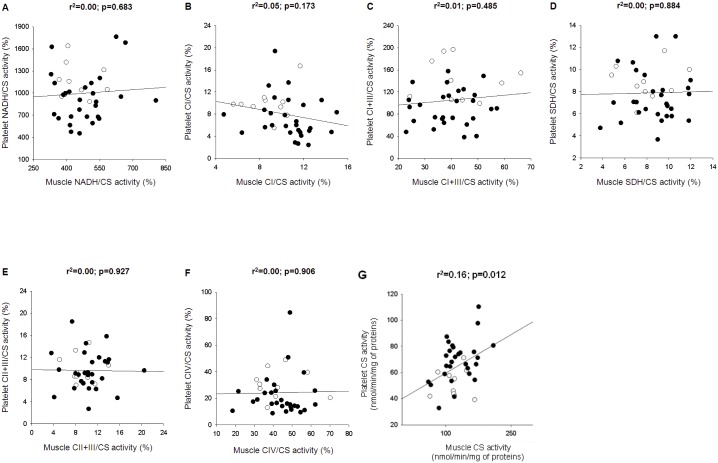
Relationship between platelet and skeletal muscle mitochondrial biochemistry. Mitochondrial biochemistry was measured on platelets (Y-axis) and triceps brachii muscle (X-axis) of ten surgical controls (white dots) and thirty patients with septic shock (<24 h from ICU admission) (black dots). Results of skeletal muscle mitochondrial biochemistry of two patients are not available due to technical troubles. Activities of nicotinamide adenine dinucleotide dehydrogenase (NADH) (**A**), complex I (CI) (**B**), complex I and III (CI+III) (**C**), succinate dehydrogenase (SDH) (**D**), complex II and III (CII+III) (**E**) and complex IV (CIV) (**F**) are expressed as percentages of citrate synthase (CS) activity (**G**). r^2^ and p values refer to Pearson product moment test.

There was no relationship between platelet and skeletal muscle respiratory chain enzyme activities in patients with septic shock (on day one) and controls (Fig. 3). Changes over time in platelets did not relate to those in skeletal muscle (Table S10).

## Discussion

This study demonstrates that mitochondrial biochemistry is variably perturbed in platelets but not in triceps brachii muscle of patients with septic shock.

In *platelets*, the activity of (respiratory chain) complex I and complex IV largely declined relative to that of (matrix) citrate synthase, that instead increased. Changes were possibly more pronounced in more severely ill patients and in non-survivors. They also tended to be worse in thrombocytopenic patients, as if mitochondrial dysfunction had accelerated platelet “death” and clearance. However, these associations should be interpreted with caution because they only emerged when variables were considered as dichotomous (low *vs.* high SOFA score; normal *vs.* abnormal platelet count). Overall, it seems that platelet respiratory chain enzymes are specifically inhibited during human septic shock and that megakaryocyte mitochondrial biogenesis and platelet mitochondrial density increase in response to that [21]. Platelet vulnerability may depend on close contact with potentially toxic circulating compounds [22]–[24] and deficiency in nuclear-encoded reactions [9]. Direct hypoperfusion should not be an issue as platelets reside in blood. One previous study reported lower platelet complex IV and higher platelet citrate synthase activities in patients with severe sepsis who finally died compared to those who did not [25]. One other study noted that platelet respiratory capacity and mitochondrial cytochrome *c* content progressively increase during fatal sepsis (further suggesting, at least, activation of mitochondrial biogenesis) [24].

In *triceps brachii muscle*, results were strikingly different. Mitochondrial biochemistry was similar to that of controls, even in patients who went on to die. Histology and histochemistry showed slight and unspecific alterations, including energy substrate depletion and non-overt inflammation. Histochemistry only seldom revealed minor-to-mild defects of complex IV activity and electron microscopy (only performed in three patients) proved unremarkable.

Absence of more significant changes in skeletal muscle mitochondria was totally unexpected. In fact, other spectrophotometric works did show inhibition of complex I and (possibly) complex IV, with or without a parallel decrease of citrate synthase activity, in vastus lateralis muscle of patients with sepsis, especially if severe or prolonged [5]–[9]. We then purposely studied patients with septic shock, usually on high dose of catecholamines (those at higher risk for mitochondrial dysfunction [6]), either on day one or seven. We performed subgroup analysis to select most severe cases and complemented biochemistry with histology, histochemistry and electron microscopy. Even so, all our efforts to detect (predicted) signs of mitochondrial dysfunction were vain.

Our present work differs from those reported above because it examined mitochondrial function in triceps brachii, and not in vastus lateralis, muscle. Muscles differ in their mitochondrial content or activity [26], susceptibility to oxidative damage [27] and blood flow [28]. As a consequence, sepsis may diversely affect different muscles, just as other systemic insults do [29]. In animals, sepsis changes mitochondrial integrity [30], [31], adenosine triphosphate levels [32] and protein turnover [33], [34] in some muscle groups more than in others. In humans, it apparently affects mitochondrial morphology in liver but not in rectus abdominis [35], triggers mitochondrial biogenesis in rectus abdominis, but not in vastus lateralis [36], induces energy failure in vastus lateralis, but not in serratus anterior, muscle [7]. Therefore discrepancy between present and past findings in skeletal muscle probably reflects compartmentalization of response to infection [37]. However, we cannot exclude that it simply indicates that skeletal muscle mitochondrial biochemistry is not, at least constantly, altered during sepsis [38].

Some aspects of this study deserve a comment. First, muscle biopsies were not immediately frozen in liquid nitrogen. We considered important to remove contaminants to measure “pure” skeletal muscle mitochondrial biochemistry. In retrospect, this was probably not a very relevant issue. In fact, results did not differ between samples processed as above and those snap-frozen (please also refer to Table S1). Moreover, changes in platelet mitochondrial biochemistry could be readily detected even if pellet preparation required approximately one hour. Second, we did not measure markers of apoptosis that, according to other authors, are associated with platelet mitochondrial dysfunction and thrombocytopenia [39], [40]. Third, our study population included only few patients with fatal septic shock, who are more likely to develop skeletal muscle mitochondrial dysfunction [6]. However, our primary end-point was to clarify whether findings in platelets can be extrapolated to other organs. Regardless of severity of disease, mitochondrial respiratory chain biochemistry was largely inhibited in platelets but not in triceps brachii muscle. Fourth, our negative results do not exclude that aspects of skeletal muscle mitochondrial physiology not presently investigated may well derange during human sepsis.

In conclusion, sepsis-induced mitochondrial changes in human platelets are not associated with those in triceps brachii muscle. Findings in blood cells (or other specific tissues) may not reflect those in other organs and therefore should not be generalized. Further studies are needed to clarify the role of mitochondrial dysfunction in the pathogenesis of human sepsis.

## Supporting Information

Figure S1 **Relationship between severity of disease and platelet mitochondrial biochemistry.** Platelet mitochondrial biochemistry (Y-axis) and sepsis-related organ failure assessment (SOFA) score (X-axis) were measured in thirty patients with septic shock (<24 h from ICU admission). Activities of nicotinamide adenine dinucleotide dehydrogenase (NADH) (**A**), complex I (CI) (**B**), complex I and III (CI+III) (**C**), succinate dehydrogenase (SDH) (**D**), complex II and III (CII+III) (**E**) and complex IV (CIV) (**F**) are expressed as percentages of citrate synthase (CS) activity (**G**). r^2^ and p values refer to Pearson product moment test.
(DOC)

Figure S2 **Relationship between platelet count and mitochondrial biochemistry.** Platelet mitochondrial biochemistry (Y-axis) and platelet count (X-axis) were measured in ten surgical controls (white dots) and thirty patients with septic shock (<24 h from ICU admission) (black dots). Activities of nicotinamide adenine dinucleotide dehydrogenase (NADH) (A), complex I (CI) (B), complex I and III (CI+III) (C), succinate dehydrogenase (SDH) (D), complex II and III (CII+III) (E) and complex IV (CIV) (F) are expressed as percentages of citrate synthase (CS) activity (G). r^2^ and p values refer to Pearson product moment test.
(DOC)

Figure S3 **Skeletal muscle mitochondrial ultrastructure during septic shock.** Electron microscopy was performed in three patients with septic shock (including one non-survivor) to confirm normal findings at biochemistry, histology and histochemistry. Mitochondria appear classically located near the Z-line, with normal morphology and ultrastructure. Original magnification: x 7000 (**A**), x 12000 (**B**, detail of A), x 30000 (**C**), x 12000 (**D**).
(DOC)

Table S1 **Skeletal muscle mitochondrial biochemistry: effect of time from biopsy to storage in liquid nitrogen.** Skeletal muscle mitochondrial biochemistry was always measured on samples that were frozen in liquid nitrogen within 10–15 min from biopsy. In five surgical controls and five patients with septic shock, one additional sample was immediately (snap) frozen in liquid nitrogen. Results obtained from samples processed in either way are reported. NADH: nicotinamide adenine dinucleotide dehydrogenase. SDH: succinate dehydrogenase. CS: citrate synthase. p values refer to paired Student’s *t* or Wilcoxon rank sum tests.
(DOC)

Table S2 **Platelet mitochondrial biochemistry in patients with septic shock and low or high sepsis-related organ failure assessment (SOFA) score.** Mitochondrial biochemistry was measured on platelets of ten surgical controls and thirty patients with septic shock (<24 h from ICU admission). Median SOFA score of patients with septic shock was 9. Patients with SOFA score ≤9 were classified as “less severe” and those with SOFA score >9 as “more severe”. NADH: nicotinamide adenine dinucleotide dehydrogenase. SDH: succinate dehydrogenase. CS: citrate synthase. p values refer to Student’s *t* or Wilcoxon rank sum tests, one-way ANOVA or ANOVA on ranks. *p<0.05 *vs.* surgical controls on post-hoc comparisons (Holm-Sidak or Dunn’s method).
(DOC)

Table S3 **Platelet mitochondrial biochemistry in patients with septic shock and low or normal platelet count.** Mitochondrial biochemistry was measured on platelets of ten surgical controls and thirty patients with septic shock (<24 h from ICU admission). Platelet count was considered normal if higher than 150*10^3^/mm^3^. NADH: nicotinamide adenine dinucleotide dehydrogenase. SDH: succinate dehydrogenase. CS: citrate synthase. p values refer to one-way ANOVA or ANOVA on ranks. *p<0.05 *vs.* surgical controls on post-hoc comparisons (Holm-Sidak or Dunn’s method).
(DOC)

Table S4 **Platelet mitochondrial biochemistry in patients with fatal or non-fatal septic shock.** Mitochondrial biochemistry was measured on platelets of ten surgical controls and thirty patients with septic shock (<24 h from ICU admission). Based on hospital outcome, patients with septic shock were classified as “survivors” or “non-survivors”. NADH: nicotinamide adenine dinucleotide dehydrogenase. SDH: succinate dehydrogenase. CS: citrate synthase. p values refer to one-way ANOVA or ANOVA on ranks. *p<0.05 *vs.* surgical controls on post-hoc comparisons (Holm-Sidak or Dunn’s method).
(DOC)

Table S5 **Platelet and skeletal muscle mitochondrial biochemistry during septic shock (day seven).** Mitochondrial biochemistry was measured on platelets and triceps brachii muscle of ten surgical controls and twenty (out of thirty) patients with (or recovering from) septic shock, seven days after ICU admission. By day seven, nine patients had already been discharged from the ICU (three deaths) and one had developed severe thrombocytopenia. On day seven, only five patients were still on catecholamine(s) and median SOFA score was 5 (3–6) (p<0.001 *vs.* median SOFA score on day one). Results of mitochondrial biochemistry of three patients are not fully available due to technical troubles. NADH: nicotinamide adenine dinucleotide dehydrogenase. SDH: succinate dehydrogenase. CS: citrate synthase. p values refer to Student’s *t* or Wilcoxon rank sum tests.
(DOC)

Table S6 **Skeletal muscle mitochondrial biochemistry in patients with septic shock and low or high sepsis-related organ failure assessment (SOFA) score.** Mitochondrial biochemistry was measured on triceps brachii muscle of ten surgical controls and twenty-eight patients with septic shock (<24 h from ICU admission). Median SOFA score of patients with septic shock was 9. Patients with SOFA score ≤9 were classified as “less severe” and those with SOFA score >9 as “more severe”. NADH: nicotinamide adenine dinucleotide dehydrogenase. SDH: succinate dehydrogenase. CS: citrate synthase. p values refer to Student’s *t* or Wilcoxon rank sum tests, one-way ANOVA or ANOVA on ranks. *p<0.05 *vs.* surgical controls on post-hoc comparisons (Holm-Sidak or Dunn’s method).
(DOC)

Table S7 **Skeletal muscle mitochondrial biochemistry in patients with septic shock and low or high central (or mixed) venous oxygen saturation.** Mitochondrial biochemistry was measured on triceps brachii muscle of ten surgical controls and twenty-eight patients with septic shock (<24 h from ICU admission). Patients with venous oxygen saturation ≥70% were classified as “normoxic” and those with venous oxygen saturation <65% as “hypoxic” (two patients with venous oxygen saturation between 65–70% were excluded from this analysis). NADH: nicotinamide adenine dinucleotide dehydrogenase. SDH: succinate dehydrogenase. CS: citrate synthase. p values refer to Student’s *t* or Wilcoxon rank sum tests, one-way ANOVA or ANOVA on ranks. *p<0.05 *vs.* surgical controls on post-hoc comparisons (Holm-Sidak or Dunn’s method).
(DOC)

Table S8 **Skeletal muscle mitochondrial biochemistry in patients with septic shock with low or high lactate blood level.** Mitochondrial biochemistry was measured on triceps brachii muscle of ten surgical controls and twenty-eight patients with septic shock (<24 h from ICU admission). Patients with blood lactate <2 mmol/l were classified as “normoxic” and those with blood lactate >5 mmol/l as “hypoxic” (eight patients with blood lactate between 2–5 mmol/l were excluded from this analysis). NADH: nicotinamide adenine dinucleotide dehydrogenase. SDH: succinate dehydrogenase. CS: citrate synthase. p values refer to Student’s *t* or Wilcoxon rank sum tests, one-way ANOVA or ANOVA on ranks. *p<0.05 *vs.* surgical controls on post-hoc comparisons (Holm-Sidak or Dunn’s method).
(DOC)

Table S9 **Skeletal muscle mitochondrial biochemistry in patients with fatal or non-fatal septic shock.** Mitochondrial biochemistry was measured on triceps brachii muscle of ten surgical controls and twenty-eight patients with septic shock (<24 h from ICU admission). Based on hospital outcome, patients with septic shock were classified as “survivors” and “non-survivors”. NADH: nicotinamide adenine dinucleotide dehydrogenase. SDH: succinate dehydrogenase. CS: citrate synthase. p values refer to one-way ANOVA or ANOVA on ranks. *p<0.05 *vs.* surgical controls on post-hoc comparisons (Holm-Sidak or Dunn’s method).
(DOC)

Table S10 **Relationship between changes over time of platelet and skeletal muscle mitochondrial biochemistry.** Mitochondrial biochemistry of platelets and triceps brachii muscle was assessed on day one and seven in fifteen patients with septic shock. Results of mitochondrial biochemistry of five patients are not fully available due to technical troubles. NADH: nicotinamide adenine dinucleotide dehydrogenase. SDH: succinate dehydrogenase. CS: citrate synthase. r^2^ and p values refer to Pearson product moment tests.
(DOC)
