# Supplementary figures and images for: Mitochondrial Changes in Platelets Are Not Related to Those in Skeletal Muscle during Human Septic Shock
Source: PLoS One. 2014 May 1;9(5):e96205. doi: 10.1371/journal.pone.0096205 (PMC4006866; doi:10.1371/journal.pone.0096205)

**Figure S1. Relationship between severity of disease and platelet mitochondrial biochemistry.**


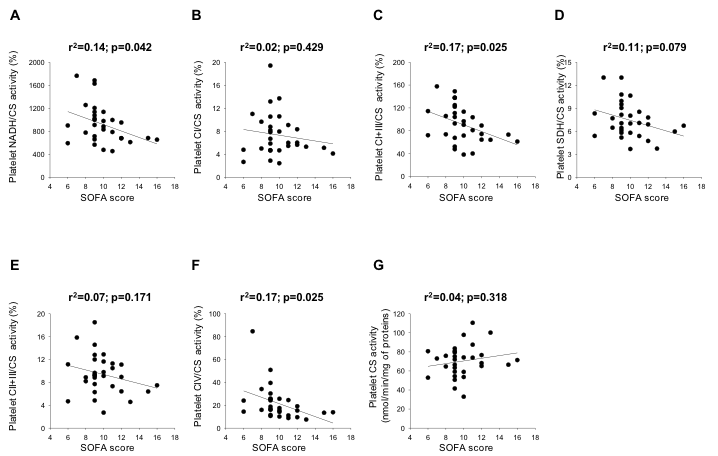

Supplement: Figure S1 — Relationship between severity of disease and platelet mitochondrial biochemistry. Platelet mitochondrial biochemistry (Y-axis) and sepsis-related organ failure assessment (SOFA) score (X-axis) were measured in thirty patients with septic shock (<24 h from ICU admission). Activities of nicotinamide adenine dinucleotide dehydrogenase (NADH) (A), complex I (CI) (B), complex I and III (CI+III) (C), succinate dehydrogenase (SDH) (D), complex II and III (CII+III) (E) and complex IV (CIV) (F) are expressed as percentages of citrate synthase (CS) activity (G). r2 and p values refer to Pearson product moment test. (DOC) [file pone.0096205.s001.doc]

**Figure S2. Relationship between platelet count and mitochondrial biochemistry.**


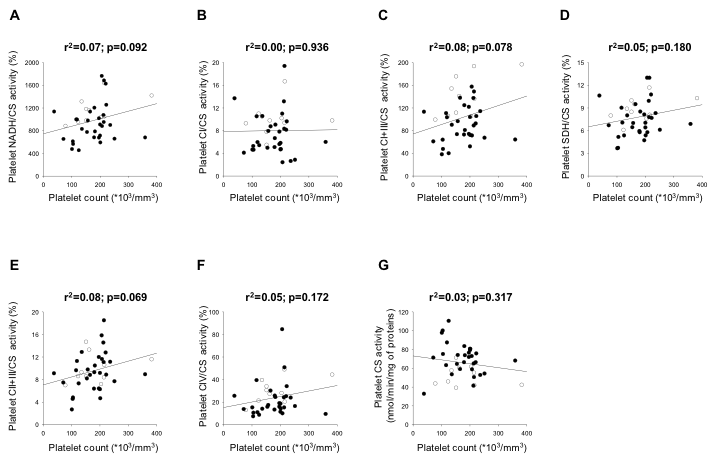

Supplement: Figure S2 — Relationship between platelet count and mitochondrial biochemistry. Platelet mitochondrial biochemistry (Y-axis) and platelet count (X-axis) were measured in ten surgical controls (white dots) and thirty patients with septic shock (<24 h from ICU admission) (black dots). Activities of nicotinamide adenine dinucleotide dehydrogenase (NADH) (A), complex I (CI) (B), complex I and III (CI+III) (C), succinate dehydrogenase (SDH) (D), complex II and III (CII+III) (E) and complex IV (CIV) (F) are expressed as percentages of citrate synthase (CS) activity (G). r2 and p values refer to Pearson product moment test. (DOC) [file pone.0096205.s002.doc]

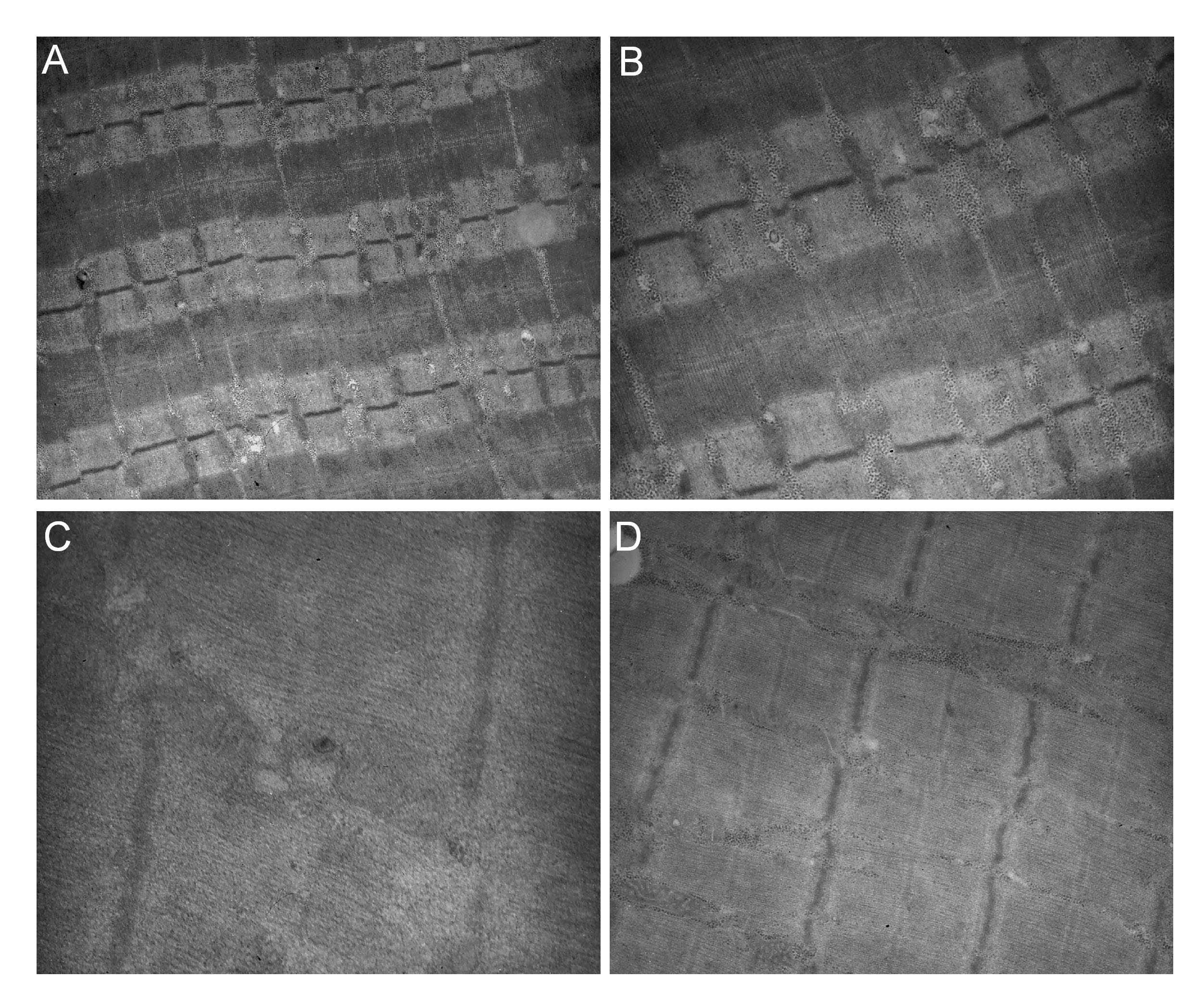
**Figure S3. Skeletal muscle mitochondrial ultrastructure during septic shock.**

Supplement: Figure S3 — Skeletal muscle mitochondrial ultrastructure during septic shock. Electron microscopy was performed in three patients with septic shock (including one non-survivor) to confirm normal findings at biochemistry, histology and histochemistry. Mitochondria appear classically located near the Z-line, with normal morphology and ultrastructure. Original magnification: x 7000 (A), x 12000 (B, detail of A), x 30000 (C), x 12000 (D). (DOC) [file pone.0096205.s003.doc]
