# Supplementary material for: Mitochondrial Changes in Platelets Are Not Related to Those in Skeletal Muscle during Human Septic Shock
Source: PLoS One. 2014 May 1;9(5):e96205. doi: 10.1371/journal.pone.0096205 (PMC4006866; doi:10.1371/journal.pone.0096205)
Supplement: Table S1 — Skeletal muscle mitochondrial biochemistry: effect of time from biopsy to storage in liquid nitrogen. Skeletal muscle mitochondrial biochemistry was always measured on samples that were frozen in liquid nitrogen within 10–15 min from biopsy. In five surgical controls and five patients with septic shock, one additional sample was immediately (snap) frozen in liquid nitrogen. Results obtained from samples processed in either way are reported. NADH: nicotinamide adenine dinucleotide dehydrogenase. SDH: succinate dehydrogenase. CS: citrate synthase. p values refer to paired Student’s t or Wilcoxon rank sum tests. (DOC) [file pone.0096205.s004.doc]

**Table S1**. **Skeletal muscle mitochondrial biochemistry: effect of time from biopsy to storage in liquid nitrogen.**

|  | **Surgical Controls** | |  | **Septic Shock** | |  |
| --- | --- | --- | --- | --- | --- | --- |
|  | Snap Frozen | Frozen after 10-15’ | p | Snap Frozen | Frozen after 10-15’ | p |
| n | 5 | 5 |  | 5 | 5 |  |
| NADH/CS (%) | 411±50 | 406±66 | 0.625 | 420±46 | 413±89 | 0.821 |
| Complex I/CS (%) | 8.1±2.7 | 9.5±1.5 | 0.108 | 10.3±2.9 | 8.7±2.6 | 0.280 |
| Complex I+III/CS (%) | 32±6 | 37±10 | 0.063 | 33±8 | 32±5 | 0.818 |
| SDH/CS (%) | 6.2±2.4 | 7.2±2.0 | 0.032 | 7.8±1.5 | 8.1±2.7 | 0.800 |
| Complex II+III/CS (%) | 6.9±2.5 | 8.0±1.7 | 0.078 | 9.6±3.0 | 10.3±4.0 | 0.544 |
| Complex IV/CS (%) | 40±6 | 42±1 | 0.685 | 42±8 | 42±13 | 0.981 |
| CS (nmol/min/mg) | 91±23 | 126±28 | 0.033 | 127±14 | 123±20 | 0.488 |
