# Supplementary material for: Mitochondrial Changes in Platelets Are Not Related to Those in Skeletal Muscle during Human Septic Shock
Source: PLoS One. 2014 May 1;9(5):e96205. doi: 10.1371/journal.pone.0096205 (PMC4006866; doi:10.1371/journal.pone.0096205)
Supplement: Table S2 — Platelet mitochondrial biochemistry in patients with septic shock and low or high sepsis-related organ failure assessment (SOFA) score. Mitochondrial biochemistry was measured on platelets of ten surgical controls and thirty patients with septic shock (<24 h from ICU admission). Median SOFA score of patients with septic shock was 9. Patients with SOFA score ≤9 were classified as “less severe” and those with SOFA score >9 as “more severe”. NADH: nicotinamide adenine dinucleotide dehydrogenase. SDH: succinate dehydrogenase. CS: citrate synthase. p values refer to Student’s t or Wilcoxon rank sum tests, one-way ANOVA or ANOVA on ranks. *p<0.05 vs. surgical controls on post-hoc comparisons (Holm-Sidak or Dunn’s method). (DOC) [file pone.0096205.s005.doc]

**Table S2. Platelet mitochondrial biochemistry in patients with septic shock and low or high sepsis-related organ failure assessment (SOFA) score.**

|  | **Surgical Controls** | **Septic Shock SOFA ≤9** | **Septic Shock SOFA >9** | **p** |
| --- | --- | --- | --- | --- |
| n | 10 | 16 | 14 |  |
| SOFA score | - | 9 (8-9) | 11 (10-12) |  |
| Platelets (*10^3^/mm^3^) | 182±83 | 192±40 | 156±78 | 0.092 |
| NADH/CS (%) | 1163±236 | 1054±379 | 770±202* | 0.005 |
| Complex I/CS (%) | 10.0±2.8 | 7.9±4.3 | 6.7±2.9* | 0.048 |
| Complex I+III/CS (%) | 142±37 | 104±34* | 76±22* | <0.001 |
| SDH/CS (%) | 8.9±1.6 | 8.2±2.5 | 6.6±1.9* | 0.025 |
| Complex II+III/CS (%) | 9.9±2.6 | 10.1±3.8 | 8.6±2.9 | 0.392 |
| Complex IV/CS (%) | 31±11 | 28±19 | 15±5* | 0.001 |
| CS (nmol/min/mg) | 52±11 | 67±13* | 74±20* | 0.005 |
