# Supplementary material for: Mitochondrial Changes in Platelets Are Not Related to Those in Skeletal Muscle during Human Septic Shock
Source: PLoS One. 2014 May 1;9(5):e96205. doi: 10.1371/journal.pone.0096205 (PMC4006866; doi:10.1371/journal.pone.0096205)
Supplement: Table S3 — Platelet mitochondrial biochemistry in patients with septic shock and low or normal platelet count. Mitochondrial biochemistry was measured on platelets of ten surgical controls and thirty patients with septic shock (<24 h from ICU admission). Platelet count was considered normal if higher than 150*103/mm3. NADH: nicotinamide adenine dinucleotide dehydrogenase. SDH: succinate dehydrogenase. CS: citrate synthase. p values refer to one-way ANOVA or ANOVA on ranks. *p<0.05 vs. surgical controls on post-hoc comparisons (Holm-Sidak or Dunn’s method). (DOC) [file pone.0096205.s006.doc]

**Table S3. Platelet mitochondrial biochemistry in patients with septic shock and low or normal platelet count.**

|  | **Surgical Controls** | **Septic Shock PLT >150** | **Septic shock PLT ≤150** | **p** |
| --- | --- | --- | --- | --- |
| n | 10 | 21 | 9 |  |
| Platelets (*10^3^/mm^3^) | 182±83 | 207±42 | 102±30* |  |
| NADH/CS (%) | 1163±236 | 997±345 | 746±251* | 0.010 |
| Complex I/CS (%) | 10.0±2.8 | 7.4±3.9 | 7.2±3.5 | 0.057 |
| Complex I+III/CS (%) | 142±37 | 98±31* | 74±30* | <0.001 |
| SDH/CS (%) | 8.9±1.6 | 7.8±2.3 | 6.6±2.4 | 0.088 |
| Complex II+III/CS (%) | 9.9±2.6 | 10.1±3.4 | 7.8±3.3 | 0.173 |
| Complex IV/CS (%) | 31±11 | 24±17 | 16±10* | 0.009 |
| CS (nmol/min/mg) | 52±11 | 68±11* | 77±25* | 0.003 |
