# Supplementary material for: Mitochondrial Changes in Platelets Are Not Related to Those in Skeletal Muscle during Human Septic Shock
Source: PLoS One. 2014 May 1;9(5):e96205. doi: 10.1371/journal.pone.0096205 (PMC4006866; doi:10.1371/journal.pone.0096205)
Supplement: Table S4 — Platelet mitochondrial biochemistry in patients with fatal or non-fatal septic shock. Mitochondrial biochemistry was measured on platelets of ten surgical controls and thirty patients with septic shock (<24 h from ICU admission). Based on hospital outcome, patients with septic shock were classified as “survivors” or “non-survivors”. NADH: nicotinamide adenine dinucleotide dehydrogenase. SDH: succinate dehydrogenase. CS: citrate synthase. p values refer to one-way ANOVA or ANOVA on ranks. *p<0.05 vs. surgical controls on post-hoc comparisons (Holm-Sidak or Dunn’s method). (DOC) [file pone.0096205.s007.doc]

**Table S4. Platelet mitochondrial biochemistry in patients with fatal or non-fatal septic shock.**

|  | **Surgical Controls** | **Septic Shock Survivors** | **Septic shock Non-Survivors** | **p** |
| --- | --- | --- | --- | --- |
| n | 10 | 25 | 5 |  |
| Platelets (*10^3^/mm^3^) | 182±83 | 177±65 | 170±56 | 0.933 |
| NADH/CS (%) | 1163±236 | 950±357 | 781±161* | 0.029 |
| Complex I/CS (%) | 10.0±2.8 | 7.8±3.8 | 5.0±2.0* | 0.013 |
| Complex I+III/CS (%) | 142±37 | 93±33* | 80±24* | <0.001 |
| SDH/CS (%) | 8.9±1.6 | 7.7±2.5 | 6.3±1.3 | 0.097 |
| Complex II+III/CS (%) | 9.9±2.6 | 9.5±3.6 | 8.8±2.8 | 0.812 |
| Complex IV/CS (%) | 31±11 | 23±16 | 14±3* | 0.018 |
| CS (nmol/min/mg) | 52±11 | 71±18* | 69±7 | 0.010 |
