# Supplementary material for: Mitochondrial Changes in Platelets Are Not Related to Those in Skeletal Muscle during Human Septic Shock
Source: PLoS One. 2014 May 1;9(5):e96205. doi: 10.1371/journal.pone.0096205 (PMC4006866; doi:10.1371/journal.pone.0096205)
Supplement: Table S5 — Platelet and skeletal muscle mitochondrial biochemistry during septic shock (day seven). Mitochondrial biochemistry was measured on platelets and triceps brachii muscle of ten surgical controls and twenty (out of thirty) patients with (or recovering from) septic shock, seven days after ICU admission. By day seven, nine patients had already been discharged from the ICU (three deaths) and one had developed severe thrombocytopenia. On day seven, only five patients were still on catecholamine(s) and median SOFA score was 5 (3–6) (p<0.001 vs. median SOFA score on day one). Results of mitochondrial biochemistry of three patients are not fully available due to technical troubles. NADH: nicotinamide adenine dinucleotide dehydrogenase. SDH: succinate dehydrogenase. CS: citrate synthase. p values refer to Student’s t or Wilcoxon rank sum tests. (DOC) [file pone.0096205.s008.doc]

**Table S5. Platelet and skeletal muscle mitochondrial biochemistry during septic shock (day seven).**

|  | **Platelet** | |  | **Skeletal muscle** | |  |
| --- | --- | --- | --- | --- | --- | --- |
|  | Surgical Controls | Septic Shock | p | Surgical Controls | Septic Shock | p |
| n | 10 | 17 |  | 10 | 18 |  |
| NADH/CS (%) | 1163±236 | 1027±388 | 0.042 | 448±80 | 525±182 | 0.240 |
| Complex I/CS (%) | 10.0±2.8 | 6.7±3.5 | 0.020 | 8.8±1.9 | 9.5±2.9 | 0.221 |
| Complex I+III/CS (%) | 142±37 | 113±48 | 0.112 | 43±12 | 37±11 | 0.186 |
| SDH/CS (%) | 8.9±1.6 | 9.0±2.6 | 0.911 | 8.0±2.2 | 8.0±2.2 | 0.511 |
| Complex II+III/CS (%) | 9.9±2.6 | 12.4±6.2 | 0.467 | 9.2±2.5 | 8.7±3.4 | 0.724 |
| Complex IV/CS (%) | 31±11 | 29±23 | 0.219 | 43±12 | 37±12 | 0.277 |
| CS (nmol/min/mg) | 52±11 | 64±8 | 0.004 | 118±30 | 122±55 | 0.841 |
