# Supplementary material for: Mitochondrial Changes in Platelets Are Not Related to Those in Skeletal Muscle during Human Septic Shock
Source: PLoS One. 2014 May 1;9(5):e96205. doi: 10.1371/journal.pone.0096205 (PMC4006866; doi:10.1371/journal.pone.0096205)
Supplement: Table S6 — Skeletal muscle mitochondrial biochemistry in patients with septic shock and low or high sepsis-related organ failure assessment (SOFA) score. Mitochondrial biochemistry was measured on triceps brachii muscle of ten surgical controls and twenty-eight patients with septic shock (<24 h from ICU admission). Median SOFA score of patients with septic shock was 9. Patients with SOFA score ≤9 were classified as “less severe” and those with SOFA score >9 as “more severe”. NADH: nicotinamide adenine dinucleotide dehydrogenase. SDH: succinate dehydrogenase. CS: citrate synthase. p values refer to Student’s t or Wilcoxon rank sum tests, one-way ANOVA or ANOVA on ranks. *p<0.05 vs. surgical controls on post-hoc comparisons (Holm-Sidak or Dunn’s method). (DOC) [file pone.0096205.s009.doc]

**Table S6. Skeletal muscle mitochondrial biochemistry in patients with septic shock and low or high sepsis-related organ failure assessment (SOFA) score.**

|  | **Surgical Controls** | **Septic Shock SOFA ≤9** | **Septic Shock SOFA >9** | **p** |
| --- | --- | --- | --- | --- |
| n | 10 | 16 | 12 |  |
| SOFA score | - | 9 (8-9) | 11 (10-12) |  |
| NADH/CS (%) | 448±80 | 469±136 | 501±71 | 0.223 |
| Complex I/CS (%) | 8.8±1.9 | 10.6±2.1 | 10.7±2.5 | 0.071 |
| Complex I+III/CS (%) | 43±12 | 37±10 | 42±9 | 0.218 |
| SDH/CS (%) | 8.0±2.2 | 8.9±2.1 | 8.3±2.3 | 0.600 |
| Complex II+III/CS (%) | 9.2±2.5 | 10.6±4.1 | 10.3±2.7 | 0.535 |
| Complex IV/CS (%) | 43±12 | 42±11 | 46±10 | 0.621 |
| CS (nmol/min/mg) | 118±30 | 129±39 | 127±33 | 0.749 |
