# Supplementary material for: Mitochondrial Changes in Platelets Are Not Related to Those in Skeletal Muscle during Human Septic Shock
Source: PLoS One. 2014 May 1;9(5):e96205. doi: 10.1371/journal.pone.0096205 (PMC4006866; doi:10.1371/journal.pone.0096205)
Supplement: Table S7 — Skeletal muscle mitochondrial biochemistry in patients with septic shock and low or high central (or mixed) venous oxygen saturation. Mitochondrial biochemistry was measured on triceps brachii muscle of ten surgical controls and twenty-eight patients with septic shock (<24 h from ICU admission). Patients with venous oxygen saturation ≥70% were classified as “normoxic” and those with venous oxygen saturation <65% as “hypoxic” (two patients with venous oxygen saturation between 65–70% were excluded from this analysis). NADH: nicotinamide adenine dinucleotide dehydrogenase. SDH: succinate dehydrogenase. CS: citrate synthase. p values refer to Student’s t or Wilcoxon rank sum tests, one-way ANOVA or ANOVA on ranks. *p<0.05 vs. surgical controls on post-hoc comparisons (Holm-Sidak or Dunn’s method). (DOC) [file pone.0096205.s010.doc]

**Table S7. Skeletal muscle mitochondrial biochemistry in patients with septic shock and low or high central (or mixed) venous oxygen saturation.**

|  | **Surgical Controls** | **SvO_2_ ≥70%** | **SvO_2_ <65%** | **p** |
| --- | --- | --- | --- | --- |
| n | 10 | 19 | 7 |  |
| SvO_2_ (%) | - | 78±4 | 60±6 |  |
| NADH/CS (%) | 448±80 | 474±124 | 511±92 | 0.365 |
| Complex I/CS (%) | 8.8±1.9 | 10.7±2.2* | 11.4±2.2* | 0.031 |
| Complex I+III/CS (%) | 43±12 | 40±10 | 40±9 | 0.669 |
| SDH/CS (%) | 8.0±2.2 | 9.0±2.0 | 8.4±2.7 | 0.503 |
| Complex II+III/CS (%) | 9.2±2.5 | 10.6±3.7 | 11.3±2.5 | 0.331 |
| Complex IV/CS (%) | 43±12 | 44±10 | 49±10 | 0.507 |
| CS (nmol/min/mg) | 118±30 | 130±38 | 128±33 | 0.699 |
