# Supplementary material for: Mitochondrial Changes in Platelets Are Not Related to Those in Skeletal Muscle during Human Septic Shock
Source: PLoS One. 2014 May 1;9(5):e96205. doi: 10.1371/journal.pone.0096205 (PMC4006866; doi:10.1371/journal.pone.0096205)
Supplement: Table S8 — Skeletal muscle mitochondrial biochemistry in patients with septic shock with low or high lactate blood level. Mitochondrial biochemistry was measured on triceps brachii muscle of ten surgical controls and twenty-eight patients with septic shock (<24 h from ICU admission). Patients with blood lactate <2 mmol/l were classified as “normoxic” and those with blood lactate >5 mmol/l as “hypoxic” (eight patients with blood lactate between 2–5 mmol/l were excluded from this analysis). NADH: nicotinamide adenine dinucleotide dehydrogenase. SDH: succinate dehydrogenase. CS: citrate synthase. p values refer to Student’s t or Wilcoxon rank sum tests, one-way ANOVA or ANOVA on ranks. *p<0.05 vs. surgical controls on post-hoc comparisons (Holm-Sidak or Dunn’s method). (DOC) [file pone.0096205.s011.doc]

**Table S8. Skeletal muscle mitochondrial biochemistry in patients with septic shock with low or high lactate blood level.**

|  | **Surgical Controls** | **Blood lactate <2 mmol/l** | **Blood lactate >5 mmol/l** | **p** |
| --- | --- | --- | --- | --- |
| n | 10 | 11 | 9 |  |
| Blood lactate (mmol/l) | - | 2 (1-2) | 8 (7-10) |  |
| NADH/CS (%) | 448±80 | 478±142 | 498±114 | 0.641 |
| Complex I/CS (%) | 8.8±1.9 | 10.6±1.4* | 11.3±2.4* | 0.018 |
| Complex I+III/CS (%) | 43±12 | 40±9 | 40±10 | 0.669 |
| SDH/CS (%) | 8.0±2.2 | 9.1±1.7 | 9.3±2.0 | 0.298 |
| Complex II+III/CS (%) | 9.2±2.5 | 10.5±2.9 | 11.1±4.5 | 0.422 |
| Complex IV/CS (%) | 43±12 | 42±8 | 50±13 | 0.271 |
| CS (nmol/min/mg) | 118±30 | 126±36 | 133±43 | 0.679 |
