# Supplementary material for: Mitochondrial Changes in Platelets Are Not Related to Those in Skeletal Muscle during Human Septic Shock
Source: PLoS One. 2014 May 1;9(5):e96205. doi: 10.1371/journal.pone.0096205 (PMC4006866; doi:10.1371/journal.pone.0096205)
Supplement: Table S9 — Skeletal muscle mitochondrial biochemistry in patients with fatal or non-fatal septic shock. Mitochondrial biochemistry was measured on triceps brachii muscle of ten surgical controls and twenty-eight patients with septic shock (<24 h from ICU admission). Based on hospital outcome, patients with septic shock were classified as “survivors” and “non-survivors”. NADH: nicotinamide adenine dinucleotide dehydrogenase. SDH: succinate dehydrogenase. CS: citrate synthase. p values refer to one-way ANOVA or ANOVA on ranks. *p<0.05 vs. surgical controls on post-hoc comparisons (Holm-Sidak or Dunn’s method). (DOC) [file pone.0096205.s012.doc]

**Table S9. Skeletal muscle mitochondrial biochemistry in patients with fatal or non-fatal septic shock.**

|  | **Surgical Controls** | **Septic Shock Survivors** | **Septic shock Non-Survivors** | **p** |
| --- | --- | --- | --- | --- |
| n | 10 | 23 | 5 |  |
| NADH/CS (%) | 448±80 | 481±121 | 491±69 | 0.658 |
| Complex I/CS (%) | 8.8±1.9 | 10.6±2.4 | 10.9±1.6 | 0.070 |
| Complex I+III/CS (%) | 43±12 | 39±11 | 40±5 | 0.565 |
| SDH/CS (%) | 8.0±2.2 | 8.7±2.1 | 8.5±2.7 | 0.725 |
| Complex II+III/CS (%) | 9.2±2.5 | 10.4±3.7 | 11.1±2.3 | 0.498 |
| Complex IV/CS (%) | 43±12 | 44±11 | 46±8 | 0.913 |
| CS (nmol/min/mg) | 118±30 | 128±37 | 129±30 | 0.758 |
