# Supplementary material for: Mitochondrial Changes in Platelets Are Not Related to Those in Skeletal Muscle during Human Septic Shock
Source: PLoS One. 2014 May 1;9(5):e96205. doi: 10.1371/journal.pone.0096205 (PMC4006866; doi:10.1371/journal.pone.0096205)
Supplement: Table S10 — Relationship between changes over time of platelet and skeletal muscle mitochondrial biochemistry. Mitochondrial biochemistry of platelets and triceps brachii muscle was assessed on day one and seven in fifteen patients with septic shock. Results of mitochondrial biochemistry of five patients are not fully available due to technical troubles. NADH: nicotinamide adenine dinucleotide dehydrogenase. SDH: succinate dehydrogenase. CS: citrate synthase. r2 and p values refer to Pearson product moment tests. (DOC) [file pone.0096205.s013.doc]

**Table S10. Relationship between changes over time of platelet and skeletal muscle mitochondrial biochemistry.**

|  | **Change in platelets *vs.***  **Change in skeletal muscle** | |
| --- | --- | --- |
|  | r^2^ | p |
| NADH/CS (%) | 0.44 | 0.007 |
| Complex I/CS (%) | 0.00 | 0.926 |
| Complex I+III/CS (%) | 0.34 | 0.022 |
| SDH/CS (%) | 0.14 | 0.163 |
| Complex II+III/CS (%) | 0.11 | 0.227 |
| Complex IV/CS (%) | 0.33 | 0.026 |
| CS (nmol/min/mg) | 0.03 | 0.535 |
